# Pogostone disrupts key virulence traits of *Candida albicans*: hyphal inhibition and biofilm suppression

**DOI:** 10.1186/s12866-026-04890-3

**Published:** 2026-03-12

**Authors:** Luyao Sun, Zihan Luo, Xingju Zou, Chen Sun, Fu Peng, Cheng Peng, Qinmei Zhou

**Affiliations:** 1 Chengdu University of Traditional Chinese Medicine, Chengdu, 611137 China; 2 West China School of Pharmacy, Sichuan University, Chengdu, 610041 China

**Keywords:** Candida albicans, Pogostone, Biofilm, Hyphae, Transcriptomic analysis

## Abstract

**Background:**

*Candida albicans* (*C. albicans*) is an opportunistic mycopathogen that clinically presents serious challenges due to its ability to cause diverse infections. High mortality rates and increased resistance to antifungals are what make this pathogen particularly concerning. Pogostone (PO) is a bioactive compound with anti-*C. albicans* activity, isolated from *Pogostemon cablin* (patchouli). Yet, there is limited research concerning the mechanism of action of PO on *C. albicans* biofilms in a detailed and thorough manner. It is for this reason that this study sought to determine the inhibitory effects of PO on *C. albicans* biofilm and hyphae.

**Results:**

PO displayed a noticeable inhibitory effect against fluconazole-resistant *Candida* species that had an MIC range of 16 to 32 µg/mL. Optical microscopy and scanning electron microscopy (SEM) observations revealed that PO effectively inhibited hyphal morphotransformation. The results obtained from XTT reduction assays, crystal violet (CV) staining, and cell surface hydrophobicity (CSH) measurements indicated that PO significantly reduces metabolic activity, biomass accumulation, and surface hydrophobicity of *C. albicans* biofilms across different growth stages. Additionally, the confocal laser scanning microscopy (CLSM) imaging further demonstrated PO’s interference in the three-dimensional architecture of *C. albicans* biofilms. Notably, this study innovatively discovered that PO can inhibit the adhesion and invasion of *C. albicans* on human vaginal epithelial (VK2/E6E7) cells. Transcriptome data showed that PO affected gene expression involved in hyphal morphogenesis, biofilm formation, and several major metabolic processes. The reduced expression of the virulence gene candidates *HWP1*, *ECE1*, *ALS3*, *TPK2*, and *EFG1* was validated by qRT-PCR. The data point towards PO interfering with several biological pathways, especially ribosomal biopathways, metabolism, and gene transcription related to biofilm formation.

**Conclusions:**

PO exerts its antifungal activity against *C. albicans* by disrupting two major virulence factors: inhibition of mycelium and biofilm. This study provides a new insight into the mode of antifungal actions of PO and thus opens newer vistas for its use in alternative therapy against conventional antifungal therapies.

## Introduction

*Candida albicans* (*C. albicans*) is a commensal fungus that commonly colonizes the oral cavity, gastrointestinal tract, and urogenital regions of healthy individuals [1]. Upon development of conditions that render the host vulnerable, including immune suppression, microbial dysbiosis, or breakdown of the mucosal barrier integrity, *C. albicans* makes a pathogenic switch from noninvasive colonization to invasive infection [2–4]. Such a transition allows the fungus to cause various clinical problems, ranging from localized superficial infections to disseminated systemic diseases such as oral candidiasis, vaginitis, and candidemia [5]. In the field of nosocomial infections, invasive candidiasis became one of the most frequent and dangerous fungal-related infections [6, 7]. Although there has been significant progress made in the discovery of antifungals, there is an unacceptably high mortality rate in invasive candidiasis, especially in susceptible patients, with rates being over 40% [8]. The current options available for combating *C. albicans* infections are mostly based on azoles, polyenes, and echinocandins [9]. Azole antifungal drugs (e.g., fluconazole) have been the most widely used antifungal drugs, and the problem of resistance to these drugs is most evident [10]. Polyene antifungal drugs (e.g., amphotericin B) have strong antifungal action, but their toxic effects on the body, and specifically renal toxicity, are considerable [11]. It underlines an imperious need for specific pharmacological measures to eliminate this problem.

Biofilm formation has been considered an important virulence mechanism in the development and progression of candidiasis [4, 12]. Such biofilms typically exhibit resistance to conventional antimicrobial therapies, effectively causing infections while resisting subsequent host immune responses and antifungal drug treatments [2, 13]. Due to the rise in the development of resistance to drugs on the part of the pathogens, there is a great need for the development of therapeutic drugs to combat infections that are a result of biofilms. Natural sources are gradually being recognized as an excellent reserve for new antimicrobial agents because they possess diversity in structure and broad-spectrum activity [14]. Various plant compounds like berberine, quercetin, and artemisinin have shown uncommon modes of action against pathogenic microorganisms [15–18]. Pogostone (PO, C_12_H_16_O_4_), a major bioactive component in the essential oil of *Pogostemon cablin* [19]. Both in vitro and in vivo studies indicated that PO has an antifungal effect on vaginal candidiasis [20, 21]. In vivo evaluation has confirmed its safety, making it a safe and efficient drug for infection treatment [22]. Although PO has demonstrated therapeutic potential, current in vitro studies on its activity against *Candida* species have been limited to determining minimum inhibitory concentrations (MIC) [20]. Comprehensive and detailed research on the precise mode of action of PO against *C. albicans* biofilms remains scarce. The present work was designed to systematically characterize the inhibitory effects of PO on *C. albicans* hyphal morphogenesis and biofilm formation and to elucidate the potential molecular mechanisms underlying PO’s anti-*Candida* activity through transcriptomic analysis. This will provide scientific support for the traditional use of PO in treating *Candida* infections, particularly biofilm-associated mucosal and systemic candidiasis.

## Materials and methods

### Chemical agents

Pogostone (PO, C_12_H_16_O_4_, purity ≥ 98.0%, Lot No. AZBI2314) was purchased from Chengdu Alfa Biotechnology Co., Ltd. (Chengdu, China). Amphotericin B (C_47_H_73_NO_17_, USP grade, 750 mg/g, Lot No. A08IS221735) was obtained from Shanghai Yuanye Bio-Technology Co., Ltd. Fluconazole (C_13_H_12_F_2_N_6_O, purity ≥ 98.0%, Lot No. AFDH1353) was purchased from Chengdu Alfa Biotechnology Co., Ltd. (Chengdu, China).

### Culture conditions of fungal strains and cell lines

A total of 11 *Candida* strains were used, including nine standard strains from the Guangdong Microbial Culture Collection Center (GDMCC) and two clinical isolates of *C. albicans* provided by Chengdu Medical College (detailed information listed in Table 2). Prior to experiments, strains were cultured on SDA at 37 °C for colony preparation. For antifungal susceptibility testing, RPMI-1640 medium (Sigma-Aldrich, USA) was buffered to pH 7.0 with MOPS. Fungal suspensions were prepared from logarithmic growth stage cells in medium, and the inoculum concentration was adjusted to 1 × 10^6^ CFU/mL.

Human Vaginal Epithelial (VK2/E6E7) Cells (Lot No. CL-1024), provided by Wuhan Procell Life Technology Co., Ltd. VK2/E6E7 is an epithelial cell line isolated in 1996 from the vaginal mucosa of a 32-year-old female patient who underwent hysterectomy due to endometriosis. Under aseptic conditions, VK2/E6E7 cells were inoculated into VK2/E6E7 cell-specific medium at 37 °C in a 5% CO₂ incubator. When cells reached 80–90% confluence, they were digested with 0.25% trypsin (containing 0.05% EDTA) and adjusted to a concentration of 1 × 10⁵ cells/mL.

### Anti-*Candida* activity assays

The antifungal activities of PO against *Candida* strains were assayed according to CLSI M27-A3 guidelines with a two-fold microdilution method [23]. Fungal suspensions (1 × 10^6^ CFU/mL) within 96-well plates were exposed to various doses of PO, Amphotericin B, and Fluconazole and incubated at 37 °C for 24 h. DMSO (≤ 0.1% v/v) served as the negative control (NC). The lowest drug concentration that completely inhibited visible growth (no turbidity observed in the well with the naked eye) was defined as the minimum inhibitory concentration (MIC).

### Growth curve determination

To further explore the anti-*C. albicans* effect of PO, the standard strain CMCC 98001 was selected for fungal growth curve analysis. Refer to the previous experimental method and make slight modifications [24]. Briefly, the fungal suspensions (1 × 10^6^ CFU/mL) were incubated with PO at doses of 2, 4, 8, 16, and 32 µg/mL and incubated in an automated growth curve analyzer (Bioscreen C, Finland) at 37 °C. DMSO (≤ 0.1% v/v) served as NC. Culture density was tracked by 600 nm absorbance measurements every 30 min for 24 h.

### Hyphal morphogenesis assay

Following the methodology of previous experiments [25], the inhibitory effect of PO on mycelial growth was observed with a light microscope. The fungal suspensions (1 × 10^6^ CFU/mL) were incubated with PO at doses of 4, 8, 16, and 32 µg/mL. DMSO (≤ 0.1% v/v) was used as NC. Culture plates were incubated for 4, 8, 12, and 24 h to analyze the hyphal growth at specified time spots. The hyphal morphology was captured using a bright field microscope (Nikon Eclipse Ti2, Japan) with 40× objective lenses.

### Morphological analysis with Scanning Electron Microscopy (SEM)

The effect of PO on hyphal morphology was further evaluated using SEM, as described previously [26]. Poly-L-lysine-coated glass coverslips were sterilized by UV irradiation for 1 h and placed in 6-well plates. Each well was inoculated with 2 mL of *C. albicans* suspension (1 × 10⁶ CFU/mL) and treated with different concentrations of PO (4, 8, 16, and 32 µg/mL) for 24 h. DMSO (≤ 0.1% v/v) served as NC. For sample preparation, the coverslips were transferred to new 6-well plates for fixation, dehydration, and critical point drying. The samples were imaged using SEM (JSM-IT700HR, Japan) at appropriate magnifications.

### Biofilm analysis methodologies

#### Establishment of biofilm model

The biofilm model was constructed based on the method recommended in Reference [27] with minor modifications. The suspension (1 × 10⁶ CFU/mL) was inoculated into poly-L-lysine-coated well plates and incubated at 37 °C for 2, 4, 8, 12, 24, 36, 48, 60, and 72 h. After washing with PBS, 50 µL of XTT working solution was added to each well and incubated at 37 °C in the dark for 3 h. The biofilm metabolic activity was quantified by measuring OD 450 nm with a spectrophotometric plate reader (Molecular Devices, USA).

#### XTT reduction assay

Following the protocol outlined in Sect. Establishment of biofilm model, biofilms at varying developmental stages (4 h, 24 h, and 48 h) were prepared. Non-adherent cells were eliminated by washing with PBS. Fresh medium (200 µL) containing graded concentrations of PO (4, 8, 16, and 32 µg/mL) was then introduced, followed by additional incubation at 37 °C for 24 and 48 h. DMSO (≤ 0.1% v/v) served as NC. The metabolic viability of *C. albicans* biofilms was subsequently quantified via the XTT reduction assay as Sect. Establishment of biofilm model described.

$$\begin{aligned}\mathrm{Biofilm}\;\mathrm{metabolic}\;\mathrm{activity}\;&=\;100\%\;\times\;(\mathrm{OD}590\;_{\mathrm{treatment}}\;-\;\mathrm{OD}590\;_{\mathrm{blank}})\;\\&/\;(\mathrm{OD}590\;_{\mathrm{NC}}\;-\;\mathrm{OD}590\;_{\mathrm{blank}})\end{aligned}$$

#### Crystal Violet (CV) staining

The biofilm quantification was performed using the CV staining method [28]. Simply put, follow the procedure described in Sect. XTT reduction assay to prepare and process the biofilm. Biofilm was fixed with 4% paraformaldehyde for 15 min, followed by staining with 0.1% CV solution for 30 min. Excess stain was cleared by PBS. The stained biofilm was then dissolved by incubation with 95% ethanol for 30 min. The biofilm biomass was quantified by measuring OD 590 nm.

$$\mathrm{Biofilm}\;\mathrm{formation}\;=\;100\%\;\times\;\mathrm{OD}590\;_{\mathrm{treatment}}\;/\;\mathrm{OD}590\;_{\mathrm{NC}}$$

#### Cell Surface Hydrophobicity (CSH) assay

The CHS of *C. albicans* was determined using the water-hydrocarbon two-phase assay [29]. Specifically, follow the procedure described in Sect. XTT reduction assay to prepare and process the biofilm. After processing, the biofilms were gently washed and resuspended. Measure the absorbance (A) of the resuspended fungal suspension at 600 nm. Subsequently, add 0.3 mL of toluene, vortex for 3 min, and allow to stand for 15 min to facilitate phase separation. Measure the absorbance (B) of the lower aqueous phase at 600 nm.

$$\mathrm{Relative}\;\mathrm{CHS}\;\%\;=\;100\%\;\times\;(\mathrm{OD_{A}}-\mathrm{OD_{B}})\;/\;\mathrm{OD_{A}}$$

#### Three-dimensional visualization using Confocal Laser Scanning Microscopy (CLSM)

To visualize biofilm structural features, confocal microscopy techniques were employed as described in the literature [30]. The Petri dishes were incubated with 4 mL of suspension (1 × 10^6^ CFU/mL) for 24 h and 48 h to allow *C. albicans* to form a biofilm. Unadhered cells were rinsed with PBS, followed by adding 4 mL of fresh medium containing PO (8, 16, 32 µg/mL) for 24 h. DMSO (≤ 0.1% v/v) served as NC. The cultures were then stained with a solution containing FUN-1 (10 µM) and Concanavalin A (Con-A, 25 µg/mL) in PBS for 45 min. The biofilm was visualized by CLSM (Olympus FV1200, Japan) after washing away the excess dye.

### Human vaginal epithelial (VK2/E6E7) cell adhesion and invasion assay

Adhesion and invasion assays were performed as previously described [31]. The VK2/E6E7 cells were cultured to the logarithmic growth phase, digested with 0.25% trypsin, and adjusted to a concentration of 1 × 10⁵ cells/mL. Each well of a 24-well plate was seeded with 1 mL of cells and incubated overnight at 37 °C in a 5% CO₂ incubator until cells adhered. Dilute *C. albicans* to 1 × 10⁸ CFU/mL and treat with PO (4, 8, 16, and 32 µg/mL) for 12 h. DMSO (≤ 0.1% v/v) served as NC. After treatment, centrifuge at 5000 rpm for 5 min to collect *C. albicans*, and adjust the fungal suspension concentration to 1 × 10⁷ CFU/mL. Subsequently, 1 mL of the cell suspension was added to VK2/E6E7 cell culture plates and incubated at 37 °C for 4 h. Following co-culture, live-cell imaging was performed using label-free optical diffraction tomography.

Adhesion assay: Following 4 h of co-culture, discard the medium and wash twice with sterile PBS. Lyse cells with 100 µL of 1% Triton X-100 for 20 min and then add 900 µL of PBS to each well to mix thoroughly. Pipette 100 µL of the dilution into 900 µL of PBS and perform serial dilutions. Finally, plate 100 µL onto solid medium and count colonies after 24 h.

$$\mathrm{Adhesion}\;\mathrm{rate}\;=\;100\%\;\times\;\mathrm{CFU}\;_{\mathrm{treatment}}\;/\;\mathrm{CFU}\;_{\mathrm{NC}}$$

Invasion assay: Following 4 h of co-culture, discard the medium and wash twice with sterile PBS. Add 200 µL of gentamicin (100 µg/mL) and continue incubation for 30 min to eliminate *C. albicans* that has not invaded the cells. Lyse cells with 100 µL of 1% Triton X-100 for 20 min and then add 900 µL of PBS to each well to mix thoroughly. Pipette 100 µL of the dilution into 900 µL of PBS and perform serial dilutions. Finally, plate 100 µL onto solid medium and count colonies after 24 h.

$$\mathrm{Invasion}\;\mathrm{rate}\;=\;100\%\;\times\;\mathrm{CFU}\;_{\mathrm{treatment}}\;/\;\mathrm{CFU}\;_{\mathrm{NC}}$$

### Transcriptomics sequencing

To clarify the mechanism of PO-mediated inhibition in *C. albicans*, transcriptomic analysis was performed on samples treated with PO (16 µg/mL) and NC samples. The high-quality RNA samples were collected and selected for cDNA library construction and sequenced on an Illumina NovaSeq 6000 platform (Lianchuan Biotechnology Co., Ltd., Hangzhou, China). The differential gene expression (DGE) analysis was conducted with the DESeq2 and edgeR software packages. The GOATools package for functional annotation of DEGs by GO enrichment analysis and KEGG pathway analysis was utilized. The adjusted p-value < 0.05 was taken as the threshold.

### Quantitative real-time PCR (qRT-PCR)

qRT-PCR was employed to measure mRNA expression of biofilm-related genes (*TPK2*, *HWP1*, *ECE1*, *ALS3*, and *EFG1*) in *C. albicans*, with the methodology described previously [32]. Total RNA was purified from samples via Trizol extraction (Shanghai Bainite Biotechnology Co., Ltd., China) and reverse-transcribed into cDNA using an ExonScript RT SuperMix with dsDNase (Chengdu Exongen Biotechnology Co., Ltd., China). The amplification was achieved with UltraStart SYBR Green qPCR Master Mix (Chengdu Exongen Biotechnology Co., Ltd.). *β-actin* functioned as the endogenous control in expression analyses. Expression analysis employed the 2^−ΔΔCt^ method with custom primers detailed in Table 1. All primers were designed and validated by Beijing GenScript Biotech Co., Ltd. (Beijing, China).

**Table 1:** Primer sequences used in qRT-PCR analysis

Genes	Primer direction	Sequence (5’-3’)
*β-actin*	Forward	GCCGGTGACGACGCTCCAAGAGCTG
	Reverse	CGTGTTCAATTGGGTATCTCAAGGTC
*TPK2*	Forward	AACAACCGCAGCAACAACTTTATCC
	Reverse	TGGAGTGATGAGGTAGCAGATTGGG
*HWP1*	Forward	GGTCCAGGTGCTTCTTCTTCTCC
	Reverse	AGACGACAGCACTAGATTCCGGA
*ECE1*	Forward	GCCATCATCCACCATGCTCCAG
	Reverse	CAGGAACAGTAGGTGCTTGGTC
*ALS3*	Forward	ACTTGTGCTGGTGGTTATTGGCA
	Reverse	TGGTGCAGTTTTGGTCAGGTAGG
*EFG1*	Forward	CCGTTGCTGCTGCTACTACTACTG
	Reverse	CACCAGACACATTACTGCCACCAC

### Statistical analysis

All data are expressed as mean ± SD. Intergroup comparisons were performed using one-way ANOVA or Student’s t-test, with *p* < 0.05 considered statistically significant.

## Results

### Anti-*Candida* activity of PO

Table 2 displays the MICs of PO, amphotericin B, and fluconazole against a collection of *Candida* strains, including standard strains and clinical isolates. All 11 tested *Candida* strains were resistant to fluconazole. The MIC range for amphotericin B was 0.2–0.4 µg/mL. PO exhibited potent antifungal efficacy against all tested *Candida* strains (*C. krusei*, *C. tropicalis*, *C. glabrata*, *C. parapsilosis*, and *C. albicans*), with MIC values ranging from 16 to 32 µg/mL. PO displayed a noticeable inhibitory response to fluconazole-resistant *Candida* species, indicating that it has prospects to become a valuable antifungal agent.

**Table 2:** The antifungal activity of PO against *Candida* spp

Strains	MIC (µg/mL)		
	PO	Amphotericin B	Fluconazole
*Candida krusei* (ATCC 6258)	16	0.2	>512
*Candida tropicalis* (ATCC 750)	32	0.4	>512
*Candida glabrata* (ATCC 22019)	32	0.4	>512
*Candida parapsilosis* (ATCC 22019)	32	0.4	>512
*C. albicans* (CMCC 98001)	16	0.2	>512
*C. albicans* (ATCC 90028)	16	0.2	>512
*C. albicans* (ATCC 90029)	16	0.2	>512
*C. albicans* (ATCC 14053)	16	0.2	>512
*C. albicans* (ATCC 10231)	16	0.2	>512
CA-1	16	0.4	>512
CA-2	16	0.4	>512

### Fungal growth curve determination

**Fig. 1:**
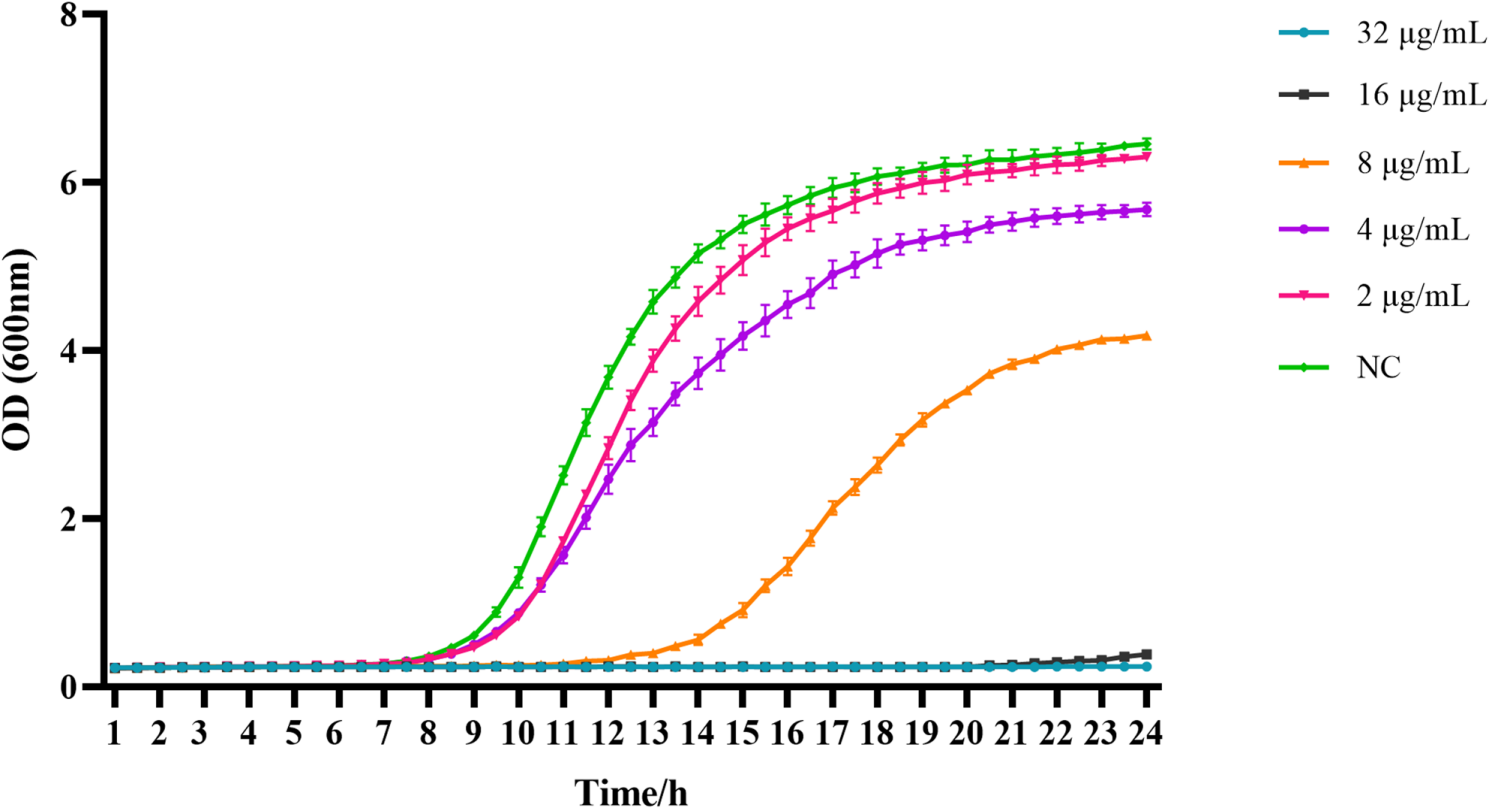
The growth curve of PO against *C. albicans*. *C. albicans* was treated with PO, and the OD 600 nm was measured every 30 min for 24 h

The antifungal activity of PO was further characterized by growth curve analysis of the *C. albicans* reference strain CMCC 98,001. As depicted in Fig. 1, the OD600 of the NC group initiated an increase at 8 h. This was followed by a period of substantial growth from 9 to 16 h. The growth curve then entered a stationary phase, which is characteristic of the organism’s pattern of growth. On the other hand, PO acted to grossly reduce the growth of *C. albicans* in a concentration-dependent manner. With the increase in the concentration of PO, the logarithmic growth phase kept delaying, which means an increased antifungal effect. PO above 16 µg/mL grossly inhibited the growth of *C. albicans*, a finding that underscores the enormous antimicrobial activity of PO against this pathogenic fungus.

### Effect of PO on hyphal growth of *C. albicans*

**Fig. 2:**
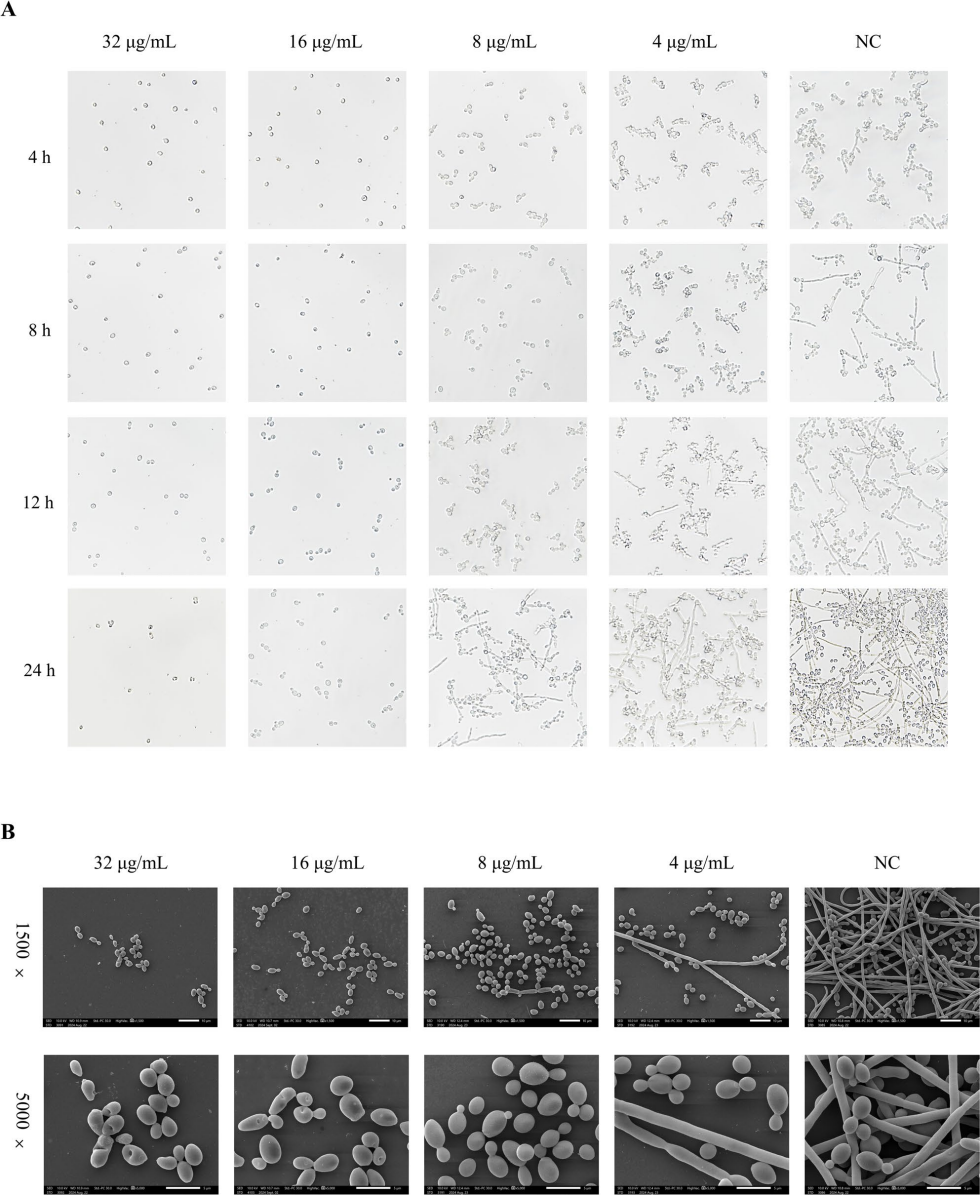
PO inhibits the hyphal growth of *C. albicans*. *C. albicans* cultures were treated with varying concentrations of PO. **A** Fungal growth observed using a 40× optical microscope after 4, 8, 12, and 24 h of treatment. **B** Fungal growth observed using a scanning electron microscope with a magnification setting of 1500× and 5000× after 24 h of treatment

Switching between yeast and hyphal morphologies represents a fundamental virulence property of *C. albicans*, one that is considered essential for biofilm formation and colonization of the host. To assess the effects of PO on the morphology of *C. albicans*, we treated fungi with varying concentrations of PO under light microscopy and SEM. As shown in Fig. 2A, the NC group had hyphal development within 4 h, and abundant mycelia were present at 24 h. On the contrary, there was significant inhibition of hyphae production by the PO treatment. Above 16 µg/ml, PO completely prevented the growth of the hyphae of the organism, with the major form of the organism being the yeast form of *C. albicans*. Inhibition of the development of hyphae due to 4 and 8 µg/ml of PO was seen to partially reduce the number and size of the hyphae. These results indicate that PO efficiently interferes in the morphological change of *C. albicans*, which is an important process for pathogenicity.

Further analysis using SEM also showed that PO inhibited the morphological transition in *C. albicans*. As can be seen in Fig. 2B, the NC group was made up of a complex of pseudohyphae, hyphae, and a few yeast cells. However, in the groups treated with PO, it was observed that there was a significant change in biofilm morphology with a reduction in hyphal elongation and an increase in the number of yeast cells. The group treated with 4 μg/mL PO was mainly composed of sparse hyphae, while those with 8 μg/mL PO were mainly composed of yeasts with minimal pseudohyphae. What was even more remarkable was that, at doses of 16 μg/mL and higher, PO not only destroyed biofilm morphology but also caused morphological abnormalities in yeasts, which were shriveled and contracted.

### The formation process of *C. albicans* biofilm

**Fig. 3:**
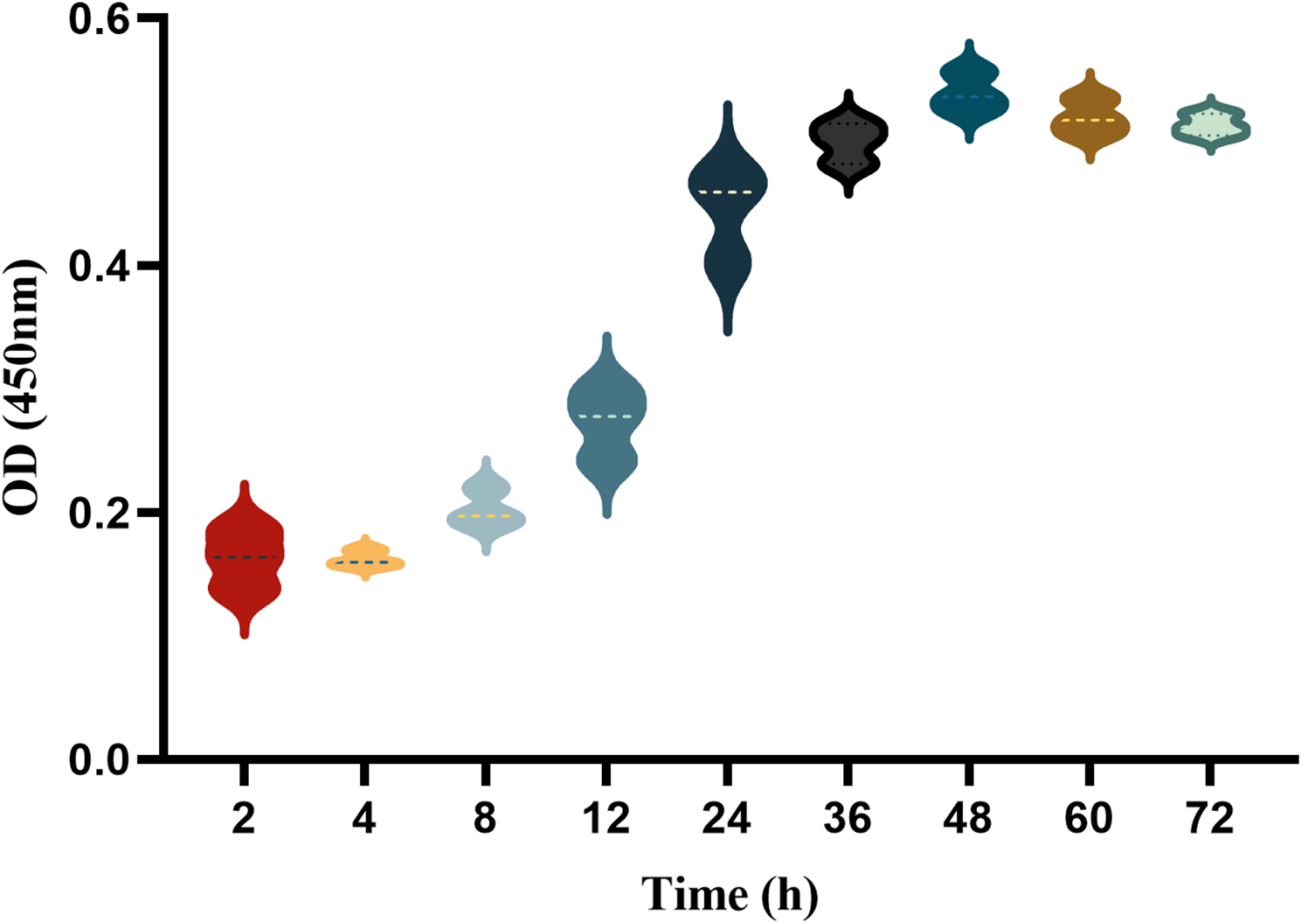
The biofilm growth curve of *C. albicans* strain CMCC 98001

There are three different stages involved in the formation of *C. albicans* biofilms, which include the initial stage (0–11 h), the intermediate stage (12–30 h), and the mature stage (31–72 h) [33]. In this study, we evaluated the biofilm-forming ability of the *C. albicans* strain CMCC 98001. As shown in Fig. 3, biofilm attachment began at 2 h, which steadily grew after 8 h. The biofilm stabilized by 24 h and peaked at 48 h, consistent with the static biofilm model established by Chen [27]. Consequently, biofilms incubated for 4 h (initial stage), 24 h (intermediate stage), and 48 h (mature stage) were chosen for further investigation.

### Effect of PO on *C. albicans* biofilm

**Fig. 4:**
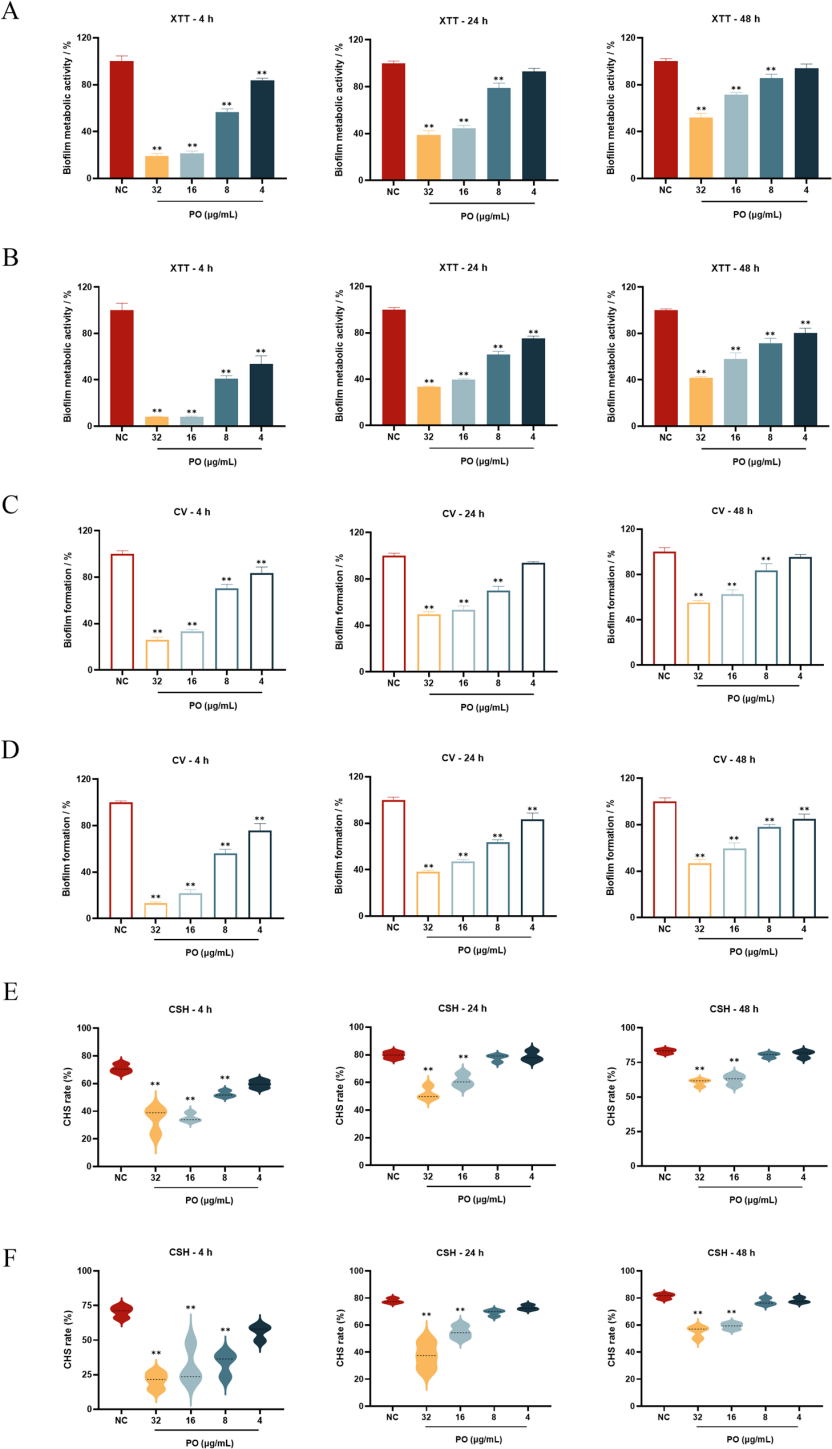
Effects of PO on *C. albicans* Biofilm. **A**,** B** XTT reduction assay measuring PO’s impact on metabolic activity of *C. albicans* biofilm at different time points (4 h, 24 h, and 48 h). PO treatment durations were 24 h (**A**) and 48 h (**B**). **C**,** D** CV staining method to assess the effect of PO on the biomass of *C. albicans* biofilm. PO treatment durations were 24 h (**C**) and 48 h (**D**). **E**,** F** Effect of PO on the hydrophobicity of *C. albicans* biofilm. PO treatment durations were 24 h (**E**) and 48 h (**F**)

XTT assay results indicated that PO significantly inhibits the metabolic activity of all growth stages of *C. albicans* biofilms. As shown in Fig. 4A, after 24-hour treatment with PO at concentrations of 32, 16, 8, and 4 µg/mL, the metabolic activity of the initial biofilm decreased by 80.95%, 78.81%, 43.60%, and 16.53%, respectively (*p* < 0.01); metabolic activity in mid-stage biofilms decreased by 61.32%, 55.65%, 21.10% (*p* < 0.01), and 7.07%, respectively; while metabolic activity in mature biofilms decreased by 47.94%, 28.47%, 14.26% (*p* < 0.01), and 5.91%. Extending the treatment duration to 48 h (Fig. 4B) resulted in significantly greater metabolic activity suppression across all biofilm stages compared to the 24-hour treatment, conclusively demonstrating PO’s sustained inhibitory effect on biofilm metabolic activity.

CV staining experiments further revealed that PO not only effectively inhibits biofilm formation but also significantly degrades mature biofilms. As displayed in Fig. 4C and D, after 24-hour PO treatment, early-stage biofilm biomass decreased by 74.10%, 66.83%, 29.74%, and 16.46% (*p* < 0.01). Mid-stage and mature biofilm biomass exhibited similar reduction trends, decreasing by 50.47%, 46.57%, 29.89% (*p* < 0.01), and 6.17% and 44.76%, 37.55%, 16.57% (*p* < 0.01), and 4.49%. A significant reduction in biofilm biomass for all stages was more prominent after 48 h of treatment compared to 24 h. These results are in tandem with the changes in metabolism noticed in the XTT assay, and together, these results establish that PO has an anti-biofilm activity, as it decreases biofilm metabolism and its biomass.

In addition, this study examined the impact of PO on the physical property changes of *C. albicans* biofilms based on CSH. As shown in Fig. 4E and F, the hydrophobicity in biofilms decreased in a concentration-dependent manner at all three phases after 24-hour and 48-hour PO treatment. Specifically, a significant reduction was found in the hydrophobicity in initial biofilms, intermediate biofilms, and mature biofilms at a 32 µg/mL and 16 µg/mL concentration of PO (*p* < 0.01). Such an outcome implies that PO could increase its anti-biofilm capacity through the reduction of microbial surface properties.

**Fig. 5:**
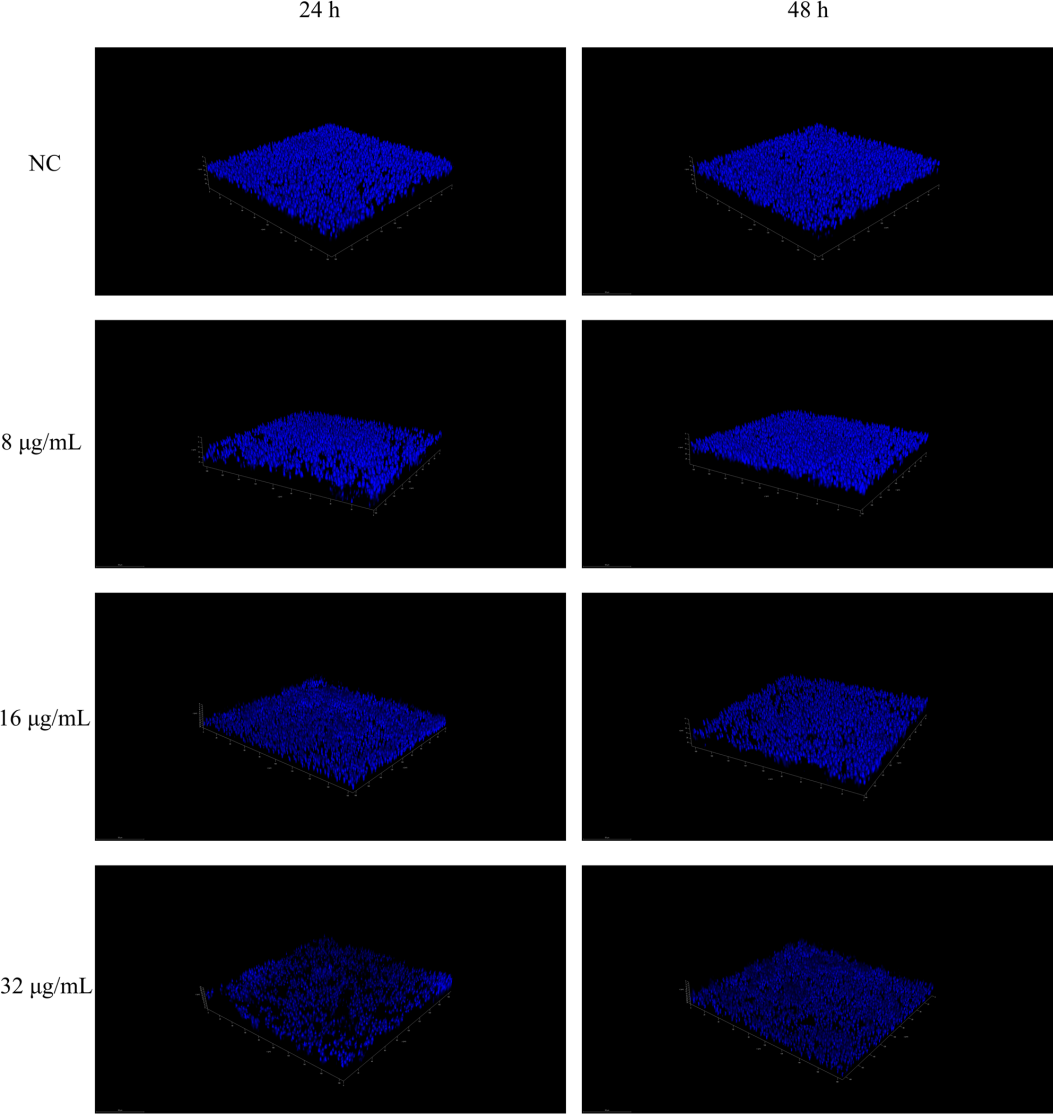
Effect of PO on the biofilm of *C. albicans*. CLSM images of *C. albicans* biofilm exposed to varying doses of PO. PO treatment durations were 24 h and 48 h

By observing using CLSM, we further confirmed the inhibitory effect of PO on *C. albicans* biofilms. From Fig. 5, it can be understood that in the NC group, *C. albicans* had formed a typical biofilm with neat architecture, thickness, and density. But in the experimental group treated with PO, there was a clear concentration gradient effect. When the concentration of PO increased, the thickness and density of *C. albicans* biofilms reduced.

### Effects of PO on *C. albicans* adhesion and invasion of VK2/E6E7 cells

**Fig. 6:**
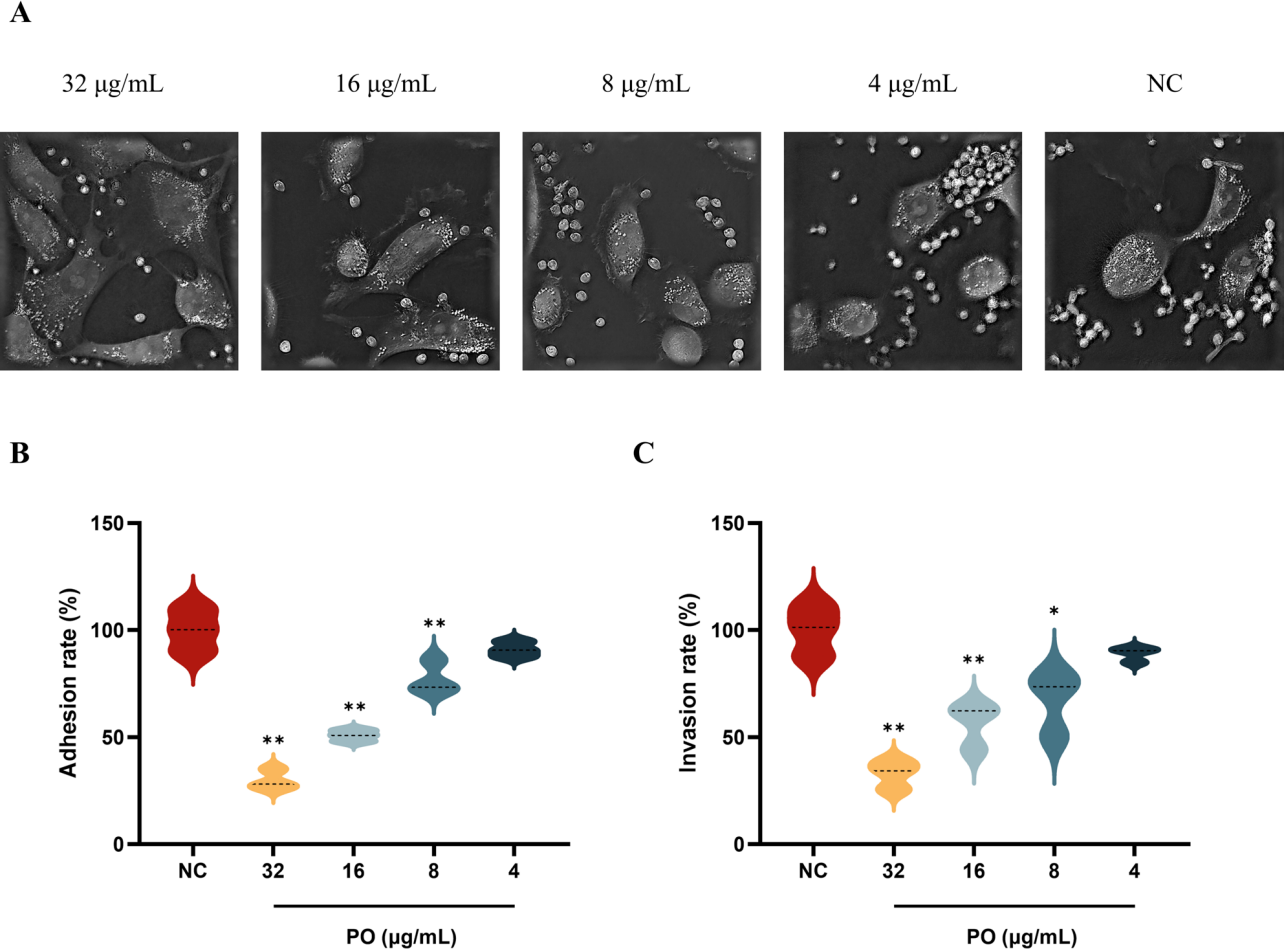
Impact of PO on *C. albicans* adhesion and invasion of VK2/E6E7 cells. **A** After 12 h of pretreatment with PO, *C. albicans* was co-cultured with VK2/E6E7 cells for 4 h, followed by live-cells imaging using label-free optical diffraction tomography. **B** The relative adhesion rate of *C. albicans* to VK2/E6E7 cells after 12 h of pretreatment with PO. **C** The relative invasion rate of *C. albicans* on VK2/E6E7 cells after 12 h of pretreatment with PO

As shown in Fig. 6A, after PO pretreatment, the ability of *C. albicans* to damage VK2/E6E7 cells decreased in a concentration-dependent manner. In the NC group, epithelial cells exhibited marked vacuolation and shrinkage, with enlarged intercellular spaces, indicating severe damage. Compared to the NC group, as drug concentration increased (4 to 32 µg/mL), VK2/E6E7 cells exhibited increasingly intact morphology, appearing more plump and well-spread, with enhanced cell density. The 32 µg/mL group demonstrated the least epithelial cell damage, indicating that PO effectively attenuates *C. albicans* invasiveness, thereby significantly protecting vaginal epithelial cells from fungal destruction.

This experiment further utilized VK2/E6E7 cells to evaluate the effect of PO on the adhesion and invasion capabilities of *C. albicans*. As shown in Fig. 6B, after 12 h of PO treatment, the proportion of *C. albicans* adhering to VK2/E6E7 cells decreased compared to the NC group. The relative adhesion rate dropped to 29.78% at 32 µg/mL, 50.69% at 16 µg/mL, and 77.18% at 8 µg/mL (*p* < 0.01). Figure 6C results indicate that the invasion rate of *C. albicans* after PO treatment was also significantly lower than the NC group. The relative invasion rate decreased to 32.79% at 32 µg/mL, 56.43% at 16 µg/mL, and 67.30% at 8 µg/mL (*p* < 0.01 or 0.05).

### Transcriptomics profiling of PO-treated *C. albicans*

#### DEGs Analysis

By comparing the transcriptome profiles between the PO-treated group and the NC group, we identified a total of 1,199 differentially expressed genes (DEGs) among 6,162 annotated genes. Specifically, 450 genes were up-regulated and 749 genes were down-regulated in the PO-treated group. Among the up-regulated genes, 23 exhibited a fold change greater than 4, while 136 of the down-regulated genes showed a fold change exceeding 4. These findings indicate that PO treatment can induce significant alterations in the gene expression profile of *C. albicans*.

The DEGs were primarily enriched in biological processes related to mycelial morphological transformation, biofilm formation, glycolysis, stress response, purine metabolism, and amino acid synthesis. Notable genes responsible for mycelial morphological transformation and biofilm formation were identified among both the highly up-regulated and down-regulated DEGs. For example, the differentially expressed genes that responded to PO treatment included *HWP1*, *ECE1*, and *ALS3*. Representative genes are shown in Tables 3 and 4 below.

**Table 3:** The representative upregulated gene list

Types	Gene Name	Description	Log2 Fold Change
Hyphae and biofilm formation	*RAS2*	Ras-related protein 2	2.906277837
	*HGT7*	Heat Shock Transcription Factor 7	3.818439763
	*SIM1*	Transcription Factor SIM1	2.018746488
	*TPK1*	cAMP-Dependent Protein Kinase 1	1.7364749
	*BRG1*	Brahma-Related Gene 1	1.255438285
	*ALS1*	Agglutinin-Like Sequence 1	2.57234856
	*FMA1*	Filamentous Growth MAPK-Associated 1	1.862266568
Purine metabolism	*HPT1*	Hypoxanthine Phosphoribosyltransferase 1	1.537103043
	*ADE5*	Adenylosuccinate Lyase	1.421569466
	*GUA1*	Guanine Phosphoribosyltransferase 1	1.51210727
	*CAALFM_C306700CA*	Adenosine Deaminase (ADA)	1.424721447
	*CAALFM_C403720CA*	Purine Permease (FUR4)	1.597928679
	*CAALFM_C204080WA*	Xanthine Dehydrogenase (XDH)	1.724250641
	*CAALFM_C305160CA*	Adenosine Kinase (ADK)	1.362562789
Amino acid metabolism	*ARO4*	Chorismate synthase	1.183316605
	*CAALFM_C603210CA*	Cysteine Synthase	1.183417406
	*CAALFM_C403740WA*	Aspartate Transaminase	1.180346692
	*LYS4*	Homocitrate synthase	1.068639601
	*CAALFM_C504910WA*	Threonine Synthase	1.07756392
	*CAALFM_C603730CA*	Aspartate kinase	1.0634608
	*CAALFM_C304370CA*	Tryptophan synthase	1.07900794

**Table 4:** The representative downregulated gene list

Types	Gene Name	Description	Log2 Fold Change
Hyphae and biofilm formation	*HWP1*	Hyphal Wall Protein 1	-4.022387875
	*ECE1*	Extent of Cell Elongation 1	-4.815985976
	*ALS3*	Agglutinin-Like Sequence 3	-2.924252849
	*EFG1*	Enhanced Filamentous Growth 1	-1.172776337
	*CAALFM_C403200CA*	Negative Regulator of Growth 1 NRG1	-1.334028488
	*CAALFM_C207770CA*	Transcription Factor for Enhanced Cell growth 1 TEC1	-1.160180835
	*CAALFM_C107080WA*	Ume6p Transcriptional Regulator 6 UME6	-1.333665378
	*PGA26*	Polysaccharide Adhesin 26	-4.148983097
	*HYR1*	Hyphal Regulated 1	-3.130808207
	*MRV5*	Mucosal Regulatory Virulence 5	-3.834150199
	*PBR1*	Polysaccharide Biosynthesis Regulator 1	-3.732973617
	*SAP5*	Secreted Aspartyl Proteinase 5	-3.494623479
	*PRA1*	Proline Rich Antigen 1	-3.037569226
	*PTP3*	Protein Tyrosine Phosphatase 3	-1.509729425
	*RIM1*	Rim1 Transcription Factor	-1.242831223
	*TPK2*	cAMP-Dependent Protein Kinase 2	-2.190543109
	*CAALFM_C102370CA*	Cyclophilin 1 CPH1	-1.236060423
Glucose metabolism	*ENO1*	Enolase 1	-1.933857492
	*TDH3*	Triosephosphate Dehydrogenase 3	-1.862607585
	*GLK1*	Glucokinase 1	-1.808082268
	*PGK1*	Phosphoglycerate Kinase 1	-2.580020537
	*PGI1*	Phosphoglucose Isomerase 1	-2.275045258
	*CAALFM_C404860WA*	Hexokinase 2 (HXK2)	-1.93219654
	*CAALFM_C503950WA*	Pyruvate kinase 1 (PYK1)	-1.463724996
	*FBA1*	Fructose-bisphosphate aldolase	-1.278745044
	*GPM1*	Phosphoglycerate mutase 1	-1.285784011
Stress response	*SOD5*	Superoxide Dismutase 5	-3.476030051
	*CAT1*	Catalase 1	-2.609715783
	*CAALFM_C405580CA*	Heat Shock Factor (HSF1)	-4.631381789
	*GST3*	Glutathione S-Transferase 3	-2.467194249
	*CAALFM_C306860CA*	Thioredoxin Reductase (TRR1)	-3.470568079
	*CAALFM_C110060CA*	Cap1 Transcription Factor	-1.144780118

#### GO enrichment analysis

**Fig. 7:**
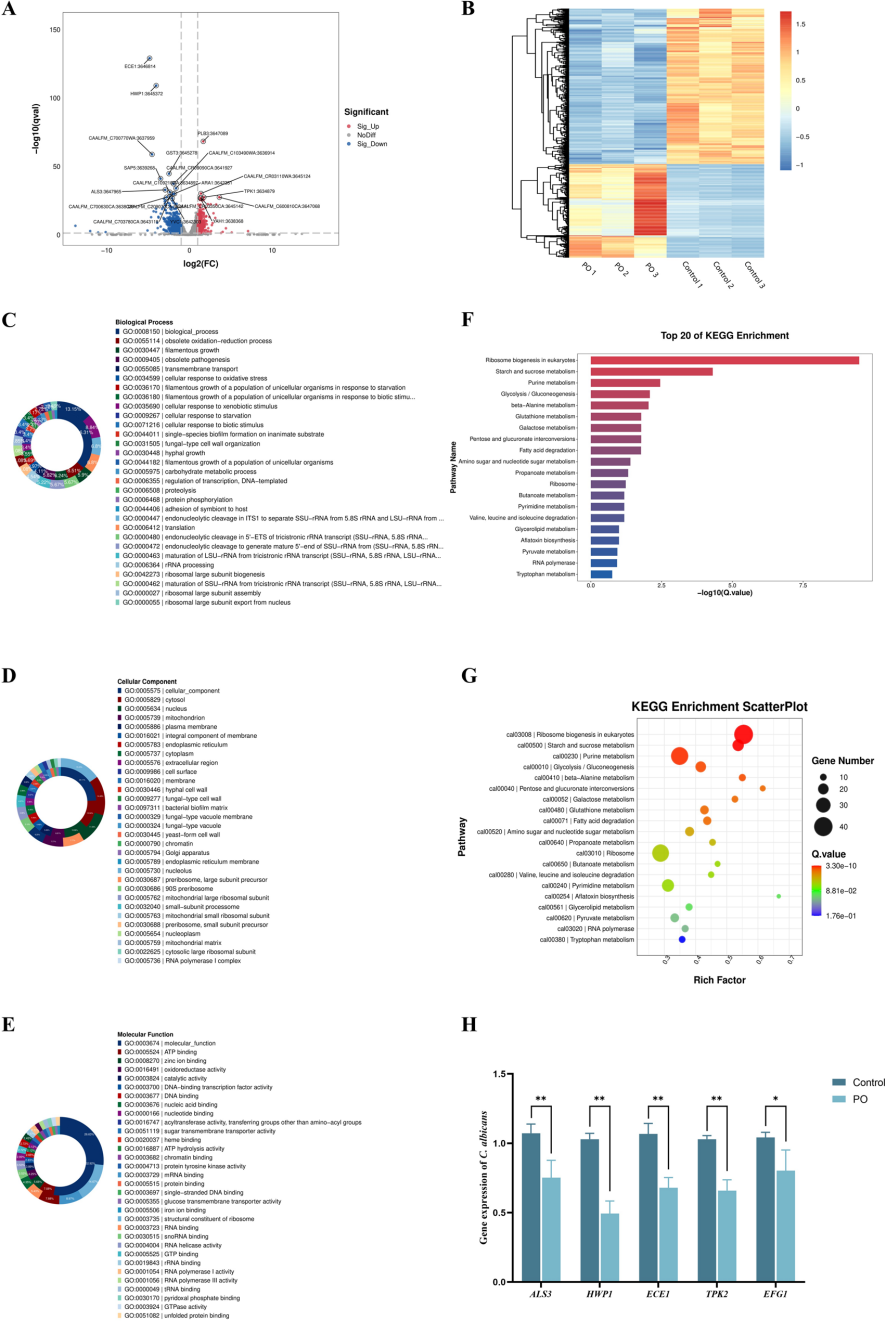
Transcriptomic analysis of PO-treated *C. albicans*. **A **Volcano plot of DEGs screen. **B** Hierarchical clustering heatmap of DEGs expression patterns. **C-E** GO enrichment analysis of DEGs. **F** The top 20 pathways with significant enrichment after PO treatment. **G** Scatterplot of KEGG pathway enrichment analysis. **H** qRT-PCR to examine the effect of PO treatment on *C. albicans* virulence gene expression

In order to systematically identify the functional properties of DEGs and examine the antimicrobial mechanism of PO, we carried out Gene Ontology (GO) analysis on the DEGs dataset. The significantly enriched keywords with a p-value < 0.05 were divided into three groups according to the ontological classification: Biological Process (BP), Cellular Component (CC), and Molecular Function (MF). Among these groups, we selected the top 30 significantly enriched keywords for each and depicted them accordingly (Fig. 7C-E).

The DEGs in the BP category (Fig. 7C) showed a remarkable enrichment for genetic information processing, including translation (GO: 0006412), ribosomal RNA processing (GO: 0006364), and biogenesis of ribosomal large subunits (GO: 0000027). More importantly, the DEGs displayed substantial enrichment in gene terms related to the development of hyphae (GO: 0036170 and GO: 0036180) and sensitivity to oxidative stress responses (GO: 0034599 and GO: 0071216). These data implicate PO as having direct antimicrobial applications via interfering with microbial morphology and redox regulation.

For the CC classification (Fig. 7D), DEGs were mainly enriched in important intracellular sites like cytoplasm (GO:0005737), nucleoplasm (GO:0005634), and mitochondria (GO:0005739), and other sites in the extracellular compartment, including biofilm (GO:0005886) and cell wall (GO:0030446). In addition, the possible relationship of DEG enrichment in the endoplasmic reticulum (GO:0005783) and Golgi apparatus (GO:0005794) with protein folding and transport in the cell should also be underscored and can be related to PO’s possible role against microbial cell wall integrity.

Concerning the MF category (Fig. 7E), the DEGs were significantly enriched in molecular functions such as RNA binding (GO:0003723), DNA binding (GO:0003677), ribonucleoprotein structural constituents (GO:0003735), catalytic activity (GO:0003824), and ATP binding (GO:0005524). The functional enrichments described above offer key hints for elucidating the mechanism of PO-mediated disruption of energy metabolism in microorganisms.

#### KEGG enrichment analysis

To illustrate the molecular mechanism of PO against *C. albicans*, we conducted KEGG pathway enrichment analysis and obtained the top 20 pathways with significant enrichment after PO treatment (Fig. 7F). Accordingly, we found that the effect of PO on ribosome function and biosynthesis significantly perturbed the two connected pathways, namely, ribosome biogenesis and ribosome pathways, which may indicate its potential inhibition of the growth of *C. albicans* by targeting the protein synthesis machinery. Because ribosomes are the site of protein synthesis, interference with ribosomal activity might limit the synthesis of important proteins, including regulators of cell cycle or enzymes of medical importance involved in the pathogenicity, which would hinder the proliferation of the pathogen *C. albicans*. Moreover, PO significantly affected carbohydrate metabolic pathways such as starch and sucrose metabolism, glycolysis/gluconeogenesis, and pentose-glucuronate interconversions. The enrichment of these pathways indicates that PO disrupts energy homeostasis by impairing the efficiency of carbon source utilization. Moreover, PO modulated amino acid metabolism. The enrichment of the branched-chain amino acid (leucine, isoleucine, and valine) degradation pathway suggests that the fungus catabolizes these amino acids into acetyl-CoA for energy compensation and releases nitrogen sources for basal metabolism. In lipid metabolism, the activation of fatty acid β-oxidation and propanoate metabolism reflects the adaptation of *C. albicans* through energy production by lipolysis and dynamic membrane lipid remodeling to mitigate the PO-induced membrane damage. At the nucleic acid level, the enrichment of the purine and pyrimidine metabolism and RNA polymerase pathway suggests that PO may directly impair DNA integrity or replication, which forces *C. albicans* to initiate a salvage of nucleotide synthesis. In summary, the multi-target effects of PO against *C. albicans* involve inhibition of protein synthesis by targeting ribosomes, disruption of energy metabolism, induction of amino acid catabolism, remodeling of lipid homeostasis, and interference with nucleic acid metabolism.

### Effect of PO on hyphal morphogenesis and biofilm-related genes in *C. albicans*

Transcriptome analysis indicated that PO significantly suppressed the expression of genes implicated in hyphal morphogenesis and biofilm formation in *C. albicans*. To validate these transcriptional changes, we conducted qRT-PCR analyses on five key virulence determinants: *ALS3* (agglutinin-like surface protein), *HWP1* (hyphal cell wall adhesin), *ECE1* (cell elongation regulator), *TPK2* (cAMP-dependent protein kinase catalytic subunit), and *EFG1* (transcription factor essential for hyphal initiation). Figure 7H reveals PO significantly reduced expression of virulence genes ALS3 (32.0%), HWP1 (53.6%), ECE1 (38.9%), TPK2 (37.1%), and EFG1 (24.0%) compared to controls (*p* < 0.01 or 0.05). The high concordance level between the transcriptional data and the qRT-PCR confirms the efficiency and robustness of our sequencing strategy. Taken together, our data show that PO inhibits the pathogenicity of *C. albicans* by interfering with the functions of major regulators for hypha formation and biofilm extracellular matrix assembly.

## Discussion

*C. albicans* is a major pathogen causing invasive candidiasis and contributes significantly to nosocomial mycoses [34]. The rising number of cases of invasive mycoses is a major contributing cause to the high mortality rates observed in the ICU [35, 36]. The rising cases of antifungal resistance in *C. albicans* exacerbate the scenario, posing major challenges to health systems worldwide [37]. The rising numbers of cases of *C. albicans* becoming resistant to traditional antifungal therapies pose major challenges in the management of public health [38]. Taking into consideration these challenges, it has become a matter of high priority to search for new antifungal compounds with novel modes of action [39]. PO, a natural compound found in patchouli, has been of interest for several years for its nontoxic and effective properties against microbial growth [22, 40, 41]. Despite the promising data obtained in vivo with PO in fighting infections, systematic in vitro studies on the action of PO against *Candida* species, particularly against *C. albicans*, have been limited [20, 21]. Therefore, biofilm formation, which is a crucial pathological process of *C. albicans*, is systematically investigated in the present study to determine the inhibitive effects of PO on the biofilms and the possible underlying mechanisms.

Through our experiments, we found that PO has good activity against various fluconazole-resistant *Candida* species, including both standard and clinically isolated, with an MIC value between 16 and 32 µg/mL. The result was in agreement with the MIC range of 3.1–50 µg/mL for PO, as reported by Li et al. [20], which shows that the use of PO could be one of the ways to combat drug-resistant infectious microorganisms. In this connection, antifungal resistance has become an important issue to be taken into account. Thus, growth curve studies further confirmed that the antifungal effect of PO on *C. albicans* is concentration-dependent.

Multiple virulence factors are employed by *C. albicans* to facilitate the process of infection; however, morphological transition and biofilm formation are the most significant [42]. This pathogen can exist in several cellular morphologies, which include yeast, pseudohyphae, hyphae, chlamydospores, and a yeast-like form [43]. Yeast-to-hyphal transition determines virulence in *C. albicans* [44, 45]. Hyphae are more invasive compared to yeast cells, which penetrate the mucosal layers and subcutaneous tissues into the bloodstream, initiating infections [46]. We have found that PO significantly retarded the hyphal development process. *C. albicans* took on predominantly yeast morphology above 16 µg/mL of PO due to complete inhibition of hyphal development. Thus, the anti-virulence activity of PO is closely related to its yeast-to-hyphal transition inhibition activity.

Biofilm formation has been proved to be important in the pathogenic, persistent, and drug-resistant nature of infection [47]. Mature biofilm may contribute to the systemic spread of *C. albicans*, in addition to decreased effectiveness of treatment, thus making it difficult to choose the right treatment strategy [48]. Consequently, *C. albicans* biofilm has been known as one of the key reasons behind increased mortality in candidiasis infection [49]. The entire process of *C. albicans* biofilm development comprises three phases: the adhesion stage (0–11 h), the proliferation stage (12–30 h), and the maturation stage (31–72 h) [33]. This study established models of *C. albicans* biofilms to assess the impact of PO upon biofilm development at critical time phases of 4 h, 24 h, and 48 h. The central issue in invasive infections caused by *C. albicans* is linked to its ability to adhere to surfaces [27]. The ability to adhere is directly linked to hydrophobicity in *C. albicans* cells that makes it easy for it to bind to both biological surfaces like human epithelial cells and non-biological surfaces like medical equipment materials [50–52]. In the current research, PO was able to decrease CSH rates of *C. albicans* and, at the same time, reduce its adhesion and invasive rates to VK2/E6E7 cells. The reduction of *C. albicans* pathogenicity by PO may be considered to be one of its main action modes. In addition, results obtained from XTT and CV staining tests revealed that PO was able to significantly decrease *C. albicans* biofilm metabolic activity and cellular biomass at three different stages. Moreover, experimental results obtained using CLSM clearly supported and verified the inhibitory effect of PO on biofilm formation and structural development. These pioneering findings clearly highlight and elaborate on the dual role of PO in targeting and fighting biofilm-related infections by inhibiting the development of new biofilm and, almost simultaneously, significantly inhibiting mature and developed biofilms. This becomes more important in medical practice for efficient treatment of biofilm-related infections, which are known to be more and more resistant to common antifungal agents.

Though we extensively assessed the inhibition of PO against *C. albicans* biofilm biomass, metabolic activity, and CSH, we did not measure the exact number of dead cells present in the biofilm by confirmative techniques such as CFU or fluorescence microscopy-based live/dead staining. Consequently, though the dramatic decrease in metabolic activity among the higher concentrations of PO is a clear indicator of cell death, the exact bactericidal or bacteriostatic property of PO against biofilm cells is yet to be ascertained. Future work will be directed towards these specific measurements to have a clear understanding of its anti-biofilm activity.

The transcriptomics profiling of PO-treated *C. albicans* indicated considerable changes in gene expressions. The list of DEGs was dominantly enriched in bioprocesses such as hyphal morphological development, formation of biofilm, glycolysis, stress response, purine metabolic process, and amino acid synthesis. The downregulated genes involved in hyphal morphological development and formation of biofilm, such as *HWP1*, *ECE1*, *ALS3*, *TPK2*, and *EFG1*, imply that the antifungal action of PO might be related to its influence on key regulatory mechanisms involved in such bioprocesses. HWP1 is responsible for the translation of the hyphal cell wall protein, which plays a vital role in hyphal adhesion and formation of biofilm [53]. *ALS3* encodes a multifunctional adhesin present on *C. albicans* cell walls and plays a critical role in adhesion, host invasion, and biofilm formation [54]. *ECE1* is a regulator of hyphal growth and biofilm formation and also encodes Candidalysin, a cytolytic peptide toxin that elicits cellular stress responses [55, 56]. *TPK2* and *EFG1* play pivotal roles in biofilm formation in *C. albicans* [57, 58]. *TPK2* is part of the cAMP/PKA signaling cascade, which phosphorylates and thereby modulates the activity of *EFG1* to control cellular processes such as hyphal growth and metabolic processes [59, 60]. *EFG1* is a major regulator at the heart of the transcriptional regulatory network that governs biofilm formation. It directly binds to the promoter regions of hypha-specific genes, including *HWP1*, *ECE1*, and *ALS3*, thereby regulating their expression and influencing hyphal morphogenesis and biofilm formation [61–63]. These genes are complex in nature and have been able to create a regulatory network for *C. albicans* that makes it able to adjust its morphology and functions according to the environmental conditions, and in return, it increases its pathogenicity. The selected DEGs, by performing qRT-PCR, are further validated to check the efficiency and accuracy of our study. The DEGs that showed downregulation, including the key regulatory genes for biofilm development, *HWP1*, *ECE1*, *ALS3*, *TPK2*, and *EFG1*, in *C. albicans*, after being exposed to PO, are validated and agreed with the data generated by transcriptomics analysis.

The GO enrichment analysis further demonstrated that DEGs were significantly enriched in terms associated with genetic information processing, hyphal development, oxidative stress response, and cellular components such as cytoplasm, nucleoplasm, mitochondria, biofilm, and cell wall. These findings suggest that PO may disrupt the normal cellular function and structural integrity of *C. albicans* through interference with a variety of cellular processes and components. KEGG pathway mapping highlights crucial molecular pathways mediating antifungal effects of PO. Since ribosome function and biosynthesis pathways were significantly disrupted, PO may inhibit the synthesis of proteins by targeting the ribosomes, hence limiting the production of key virulence-associated proteins. Moreover, perturbation of carbohydrate metabolic networks, including glycolysis/gluconeogenesis and pentose-glucuronate interconversion pathways, suggests that PO disrupts energy homeostasis through impairing efficiency of utilization of carbon sources. The regulation of amino acid metabolism and lipid homeostasis is a further testament to the multi-target action mechanism of PO, not only a translation inhibitor but also a disruptor of energy metabolism and a trigger for *C. albicans*’ metabolic adaptation. The decrease in purine and pyrimidine metabolism and RNA polymerase gene regulation affects nucleic acid metabolism and points to a potential direct damage effect on *C. albicans*’ DNA integrity or replication, obliging the pathogen to activate nucleotide synthesis via salvage pathways. While the research system allowed the effects of PO on known antifungal target pathways to be elucidated, to date, no new direct molecular target has been found to be clearly defined on the basis of transcriptional analysis. Thus, one might speculate about the multi-target nature of PO, whose cumulative effect may be achieved through a synergistic inhibitory action on several targets. Future analysis should thus combine proteomics and target fishing approaches to define direct targets.

It is worth noting that a recent study [64] similarly used transcriptomics to report that butanol strongly represses the hyphae and biofilms of *C. albicans* by interfering with pathways such as ribosomal biosynthesis. This finding agrees with the core conclusion of our study regarding the anti-virulence phenotype. However, this study and our study are quite different in intensity of action and degree of response of molecular targets. Anand et al. reported a greater downregulation of major hyphal genes such as *HWP1* and *UME6*, which was most likely due to the higher butanol concentrations they used (1% v/v) in a strongly inducing environment of whole human serum, resulting in greater disruption of transcription. On the contrary, the PO used in this study showed pronounced mycelium and biofilm inhibition at much lower microgram concentrations in standard medium and thus may possess a higher degree of specificity or different initial targets. This difference suggests that PO—a naturally derived antitoxic agent—may act through a distinct mechanism from that of mere solvent stress (e.g., that caused by butanol) and points to new opportunities for developing more specific and less toxic antifungal candidates.

The results of the study are of prime importance in the field of clinical research in the quest for novel pharmacological agents targeting biofilm-related infections. Its effectiveness in suppressing the growth of the hyphal form and the biofilm formation makes it a promising drug candidate in the quest for the resolution of antimicrobial resistance in infectious diseases. The multitarget mechanism of PO in the disruption of protein synthesis, energy metabolism, and nucleic acid metabolism makes it a potential candidate in the treatment of a wide range of infections caused by *Candida*.

## Conclusions

This work offers convincing evidence that PO possesses significant efficacy in inhibiting important virulence factors in *C. albicans*, such as morphology and biofilm formation. The multi-target properties of PO, such as inhibiting protein synthesis, energy metabolism, and nucleic acid metabolism, emphasize its potential to be a new antifungal agent for fighting biofilm-causing infections.

## Data Availability

The RNA sequencing data are available via the National Center for Biotechnology Information (NCBI) with BioProject accession PRJNA1266367.
